# Postoperative C-reactive protein kinetics predict postoperative complications in patients treated with cytoreductive surgery and hyperthermic intraperitoneal chemotherapy for peritoneal carcinomatosis

**DOI:** 10.1186/s12957-020-02081-6

**Published:** 2020-11-26

**Authors:** Antoine El Asmar, Melissa Bendavides, Michel Moreau, Alain Hendlisz, Amélie Deleporte, Maher Khalife, Vincent Donckier, Gabriel Liberale

**Affiliations:** 1 Department of Surgical Oncology, Institut Jules Bordet, Université Libre de Bruxelles, 121, Blvd. de Waterloo, 1000 Brussels, Belgium; 2 Department of Statistics, Institut Jules Bordet, Université Libre de Bruxelles, Brussels, Belgium; 3 Department of Medical Oncology, Institut Jules Bordet, Université Libre de Bruxelles, Brussels, Belgium; 4 Department of Anesthesiology, Institut Jules Bordet, Université Libre de Bruxelles, Brussels, Belgium

**Keywords:** Peritoneal carcinomatosis, HIPEC, C-reactive protein, Postoperative complications, Prediction

## Abstract

**Background:**

Relatively high morbidity rates are reported after cytoreductive surgery (CRS) with hyperthermic intraperitoneal chemotherapy (HIPEC). However, early predictors of complications after CRS plus HIPEC have not been identified. The aim of this study was to evaluate the predictive role of early postoperative serum C-reactive protein (CRP) level (Day 2–4) for the detection of post-operative complications.

**Patients and methods:**

We performed a retrospective study including 94 patients treated with complete CRS (R1) and HIPEC for PC from various primary origins (2011–2016). Post-operative complications were recorded. The values for postoperative inflammatory markers (white blood cells [WBC] and platelet counts, CRP) were compared between the different groups.

**Results:**

CRP on post-operative days 2–4 was significantly higher in patients with than without complications (124 mg/L vs 46 mg/L; p < 0.0001) and higher in those with more major complications (162 mg/L vs 80 mg/L; p < 0.0012). WBC and platelet counts showed no difference within 5 days postoperatively.

**Conclusion:**

CRP levels, and kinetics mainly, between post-operative day 2 and 4, are decisive predictive markers of early and late post-operative complications after CRS plus HIPEC. The presence of post-operative complications should be suspected in patients with a high CRP mean, and a plateau level (days 2–4).

## Introduction

The use of cytoreductive surgery (CRS) with hyperthermic intraperitoneal chemotherapy (HIPEC) is becoming more and more widespread in the treatment of peritoneal carcinomatosis (PC) in multiple types of cancer [1–3].

Patients with peritoneal carcinomatosis have a dismal prognosis, irrespective of the primary tumor site, with a median survival of 6 months in the absence of treatment [4]. An estimated 66% of PCs arise from gastrointestinal (GI) tumors (colorectal (60%), gastric (20%), and pancreatic (20%) and 33% arise from tumors of other origins, including ovarian tumors in more than 50% of cases [5]. CRS with or without HIPEC has been reported to be beneficial in terms of outcomes in patients treated for PC from various origins including gastric, ovarian, pseudomyxoma peritonei (PMP), and colorectal cancer, with a median survival of 42 months in patients with PC from colorectal origin, versus 15 months for chemotherapy-only patients, and a 5-year survival of 34.5% [6–9].

However, CRS alone or combined with HIPEC may be accompanied by postoperative complications in 30% to 60% of cases [8–11], these being mainly related to the extent of CRS, the general condition of the patient, and the experience of the center [12].

Early detection of postoperative complications is important to avoid delays in management. However, in patients treated with CRS and HIPEC, the clinical detection of these complications is not always easy [13]. C-reactive protein (CRP) has been reported as a potential tool for predicting postoperative complications [14–16]. The normal level of CRP is less than 0.8 mg/L and varies beyond this limit during an inflammatory event. In general, the concentration doubles every 8 h and reaches its maximum value after 36-50 h [17]. The intensity of the inflammatory event influences the amount of CRP produced [18]. Very few data are available regarding the value of CRP measurement in patients treated with CRS and HIPEC [19].

The primary objective of this study is to evaluate whether postoperative CRP levels are predictive of postoperative complications in patients treated for PC with CRS and HIPEC.

## Methods

We performed a retrospective monocentric study, including patients treated with curative intent, with R0/R1 resections plus CRS and HIPEC, for PC of colorectal, appendicular, ovarian, and gastric cancers, between January 2011 and December 2016.

### Data Retrieval

Patient characteristics, type of surgery performed, postoperative complications, CRP and WBC values were collected from a prospective database on patients treated with CRS and HIPEC and from the institution’s program (Oribase).

### Complications

Post-operative complications, that appeared on postoperative day (POD) 4 till postoperative day 30, were recorded, and classified according to Clavien-Dindo classification (CDC).

Patients were categorized into two main groups: those who developed postoperative complications versus those who didn’t. Those who developed complications were further subdivided into subgroups, according to A- The severity of their complications: minor (I and II) versus major (III and IV), using the CD classification, and B- The nature of their complications: infectious versus non-infectious.

### Inflammatory markers

Patients receiving CRS with HIPEC are subject to preoperative laboratory work-up, including CRP levels, white blood cell (WBC) and platelet counts, and daily biomarker control during their stay in the intensive care unit (POD 0 to POD 7), including CRP levels, WBC and platelet counts. The values of postoperative inflammatory markers: WBC count, platelet count, and CRP levels, were compared between the different groups.

### Statistical analysis

CRP means and medians were calculated for the first seven days postoperatively, and the CRP average was calculated, for both, main groups and subgroups, from POD 2 until POD 4. Student’s t-test was used for the analysis and comparison of CRP averages between the two main groups (with versus without complications), and between the two subgroups (minor versus major complications). An ANOVA parametric test was used as well, to compare between patients without complications and subgroups A & B. Whenever this test revealed significant associations, a Chi-square test was done to confirm the independence of the variable. A multivariate analysis was further performed, confuting the PCI as a confounder, affecting the CRP statistical correlation with postoperative complications.

To evaluate the predictive value of postoperative CRP on the occurrence of complications, we also calculated the Positive Predictive Value (PPV) and Negative Predictive Value (NPV), Sensitivity (Se), and specificity (Spe) of the test. The CRP thresholds used for the measurements of PPV, NPV, Se, and Spe were the p50 (median) and the p25.

The effect of other inflammatory markers (WBC and platelets), on the occurrence of postoperative complications, was evaluated by calculating their averages among patients with and without postoperative complications.

## Results

A total of 94 patients were included, 66 females and 28 males. The mean age was 55 years and 6 months (median 57 years, range: 31–75). Fifty-five patients had peritoneal carcinomatosis of colorectal origin, 18 of ovarian origin, 14 of appendiceal origin, 5 of gastric origin, and 2 patients had malignant mesothelioma (Table 1). The average PCI was 10.74 (median = 8, range: 0–35).

**Table 1:** Patient characteristics, with and without post-operative complications

	Total N of patients *N* = 94 (100%)	Without complications *N *= 28 (29.8%)	With complications *N*= 66 (70.2%)
**Age (years)**			
** Mean**	55.5	54	56.2
** Median**	57	54	58
**Gender**			
** Males**	28 (29.8%)	8 (28.6%)	20 (30.3%)
** Females**	66 (70.2%)	20 (71.5%)	46 (69.7%)
**Primary cancer**			
** Colorectal**	55 (58.5%)	16 (57.1%)	39 (59.1%)
** Ovarian**	18 (19.2%)	7 (25%)	11 (16.7%)
** Pseudomyxoma**	14 (14.9%)	3 (10.7%)	11 (16.7%)
** Gastric**	5 (5.3%)	1 (3.6%)	4 (6%)
** Mesothelioma**	2 (2.1%)	1 (3.6%)	1 (1.5%)
**PCI**			
** < 6**	39 (41.5%)	15 (53.6%)	24 (36.4%)
** 7–12**	19 (20.2%)	5 (17.9%)	14 (21.2%)
** 13–16**	7 (7.5%)	2 (7.1%)	5 (7.6%)
** > 17**	25 (26.6%)	4 (14.3%)	21 (31.8%)
** Undetermined**	4 (4.2%)	2 (7.1%)	2 (3%)
**Surgical Intervention**			
** Colorectal resection**	65 (69.1%)	17 (60.7%)	48 (72.7%)
** Enterostomy**	35 (37.2%)	5 (17.9%)	29 (43.9%)
** Gastrectomy**	10 (10.6%)	2 (7.1%)	8 (12.1%)
** Cholecystectomy**	60 (63.8%)	15 (53.6%)	45 (68.2%)
** Appendectomy**	23 (24.5%)	5 (17.9%)	18 (27.3%)
** Omentectomy**	66 (70.2%)	20 (71.4%)	45 (68.2%)
** Peritonectomy**	56 (59.6%)	14 (50%)	42 (63.6%)
** Splenectomy**	27 (28.7%)	7 (25%)	20 (30.3%)
** Oophorectomy/ Hysterectomy**	36 (38.3%)	13 (46.4%)	23 (34.9%)
** Lombo-aortic lymphadenectomy**	15 (16%)	5 (18%)	10 (15.2%)
** Diaphragmatic resection**	30 (32%)	7 (25%)	21 (31.8%)

Sixty-six out of 94 patients (70.21%) developed at least 1 postoperative complication through POD 30. Table 2 provides details on the type of complications, and the number and proportion of each complication in relation to all the complications recorded in the 94 individuals. A total of 137 complications were classified under 13 categories according the CD scoring system. Thirty-nine out of 94 patients (41.49%) had a grade II complication (Table 2).

**Table 2:** Clavien-Dindo classification of complications in all patients

	*N (%)*	*Grade 1*	*Grade 2*	*Grade 3a*	*Grade 3b*	*Grade 4a*	*Grade 4b*	*Grade 5*
Anastomotic leak	**4 (2.92%)**	2 (50%)			2 (50%)			
Peritonitis	**15 (10.94%)**		12 (80%)	2 (13.33%)	1 (6.67%)			
Hemoperitoneum	**5 (3.65%)**	1 (20%)		1 (20%)	3 (60%)			
Small bowel obstruction	**3 (2.19%)**		1 (33.33%)		2 (66.67%)			
Urinary tract infection	**35 (25.55%)**	1 (2.86%)	32 (91.42%)	1 (2.86%)	1 (2.86%)			
Septicemia	**14 (10.22%)**		11 (78.57%)				2 (14.29%)	1 (7.14%)
Pancreatitis	**4 (2.92%)**	2 (50%)	1 (25%)		1 (25%)			
Cerebro-vascular accident	**3 (2.19%)**	1 (33.33%)						2 (66.67%)
Pulmonary complications	**27 (19.71%)**	4 (14.81%)	12 (44.44%)	3 (11.11%)		7 (25.94%)		1 (3.70%)
Cardio-vascular complications (including deep vein thrombosis, pulmonary embolism…)	**8 (5.84%)**		7 (87.5%)			1 (12.5%)		
Renal failure	**11 (8.03%)**	5 (45.45%)	5 (45.45%)	1 (9.10%)				
Wound infection	**8 (5.84%)**	4 (50%)	3 (37.5%)	1 (12.5%)				
Total number of complications	137	20 (14.60%)	84 (61.31%)	9 (6.57%)	10 (7.30%)	8 (5.84%)	2 (1.46%)	4 (2.92%)
Percentage of each complication in 94 patients		2.13%	41.49%	6.38%	10.64%	5.32%	1.06%	3.19%

The highest rate of postoperative complications was detected in Gastric cancers (80%), followed by Pseudomyxoma Peritonei (79%) then Colorectal (71%) and Ovarian (61%). Furthermore, the higher the PCI, the higher was the complication rate: 84% in PCIs > 17, 73% in PCIs between 7 and 16, and 62% in PCIs < 6.

Mean CRP on POD 2–4 (Table 3) was significantly lower for patients without complications compared to that of patients with complications (45.79 mg/L and 124.3 mg/L, respectively, *p* < *0.0001*). Mean CRP on POD 2–4 for patients with minor complications was also significantly lower than that of patients with major complications (80.06 mg/L and 162.4 mg/L, respectively, *p* = *0.0012*). CRP values on POD 2–4, were, therefore, higher in patients with complications, particularly for those with grade III-IV complications. The sensitivity, specificity, PPV, and NPV of postoperative CRP levels for the prediction of postoperative complications were, respectively, 85.25%, 46.17%, 78.79%, 57.14% (Table 3).

**Table 3:** CRP and WBC means between POD 2 and 4, with respect to patients’ complications status, and their respective clinical significance when present (*p* < 0.05). The PPV, NPV, Se, and Spe for the CRP thresholds p50 (median) and p25, are also shown

Patients Characteristics	WBC Mean between POD 2 and POD 4	*p*-value	CRP Mean between POD 2 and POD 4	*p*-value	p50 (cut-off = 106.1)	p25 (cut-off = 56.5)
**Without Complications**	8.23		45,79		Se: 65.57%	Se: 85.25%
**vs**		*p* > 0,05		*p* < 0,0001	Spe: 46.15%	Spe: 46.17%
**With Complications**	9.46		124,3		PPV:67.44%	PPV: 78.79%
					NPV:66.67%	NPV: 57.14%
**Without Complication**			45,79			
**vs**						
**CD 1–2**	N/A	N/A	80,06	*p* < 0,0001	N/A	N/A
**vs**						
**CD 3–4**			162,4			
**Without Complications**	8.23		45,79		Se: 57.1%	Se: 85.7%
**vs**		*p* > 0,05		*p* = 0,0004	Spe: 78.6%	Spe: 53.6%
**Infectious Complications**	9.2		107,0		PPV: 76.9%	PPV: 69.8%
					NPV: 59.5%	NPV: 75.0%
**Without Complications**	8.23		45,79		Se: 33.3%	Se: 100%
**vs**		*p* > 0,05		*p* = 0,0163	Spe: 78.6%	Spe: 53.6%
**Non-Infectious Complications**	9.8		107,5		PPV: 14.3%	PPV: 18.8%
					NPV: 91.7%	NPV: 100%

(*CRP* C-reactive protein, *WBC* White blood cells, *POD* Postoperative day, *PPV* Positive Predictive Value, *NPV* Negative Predictive Value, *Se* Sensitivity, *Spe* Specificity)

When compared, WBC and platelets levels did not show statistical significance, in predicting whether the patient will develop a complication, particularly between POD 2–4.

## Discussion

Early postoperative (POD 2–4) CRP levels of patients treated for PCs from different origins by cytoreductive surgery and HIPEC predict postoperative infectious and non-infectious complications. Conversely, WBC and platelet counts are not predictive of postoperative complications in these patients.

The morbidity rate of patients treated with CRS and HIPEC for PC reported in the literature is around 30%-60% [8]. Ours was around 70%, including the CD I rated complications. Our study showed as well a higher rate of postoperative complications in gastric cancer, pseudomyxoma peritonei and high PCIs, correlating with available literature data [20].

In practice, the diagnosis of early post-operative complications in patients who have undergone multiple intestinal resections and anastomoses, multiple organ resections, peritonectomies, or diaphragmatic resection is often difficult. A biological marker that could allow for earlier detection of complications, and thus, an earlier management of patients likely to develop postoperative complications, would be a very useful tool for improving the management of these patients.

CRP is a commonly used biological marker, being accurate, inexpensive, and easy to measure. CRP levels reflect the intensity of an inflammatory reaction, and CRP also participates in activating the inflammatory cascade [8]. Recent studies have shown its efficacy for predicting postoperative complications, particularly in abdominal surgery [16, 21].

However, its usefulness in the setting of CRS combined with HIPEC has not been determined yet. To our knowledge, only one study has focused on the value of CRP for predicting postoperative complications of patients treated with CRS and HIPEC [21]. Fernandez et al. showed that CRP levels at POD 2 were highly associated with postoperative early infectious complications in patients who underwent CRS plus HIPEC for PC of ovarian origin [22]. However, they did not prove its significance in predicting non-infectious complications or late infectious ones.

**Fig. 1:**
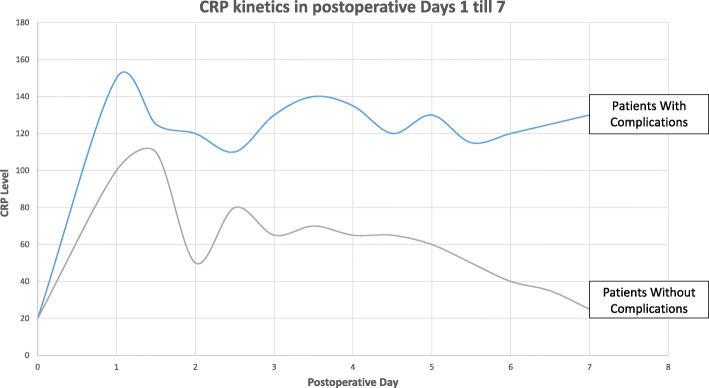
shows the CRP kinetics for patients without postoperative complications versus those who developed complications, between POD 2 and POD 4

In our study, we observed that CRP levels were higher in patients who developed postoperative complications whether infectious or not (Table 1). Moreover, higher CRP levels reflected a higher complication grade (Table 3). Also, we would like to emphasize that the early kinetics of CRP levels, rather than the actual CRP value, is more important. The sensitivity of detecting complications using CRP kinetics between POD 2 & 4 was 85%, with a specificity of 45%, PPV of 79%, and NPV of 57%. As shown in Fig. 1, the patients who were more prone to develop postoperative complications were those in whom CRP levels tended to rise and maintain a certain plateau between POD 2 and 4. When comparing the curves, significant difference in CRP levels exists after POD 5 between the 2 patients’ categories, however, even though CRP levels are clearly higher in the complications group, the “slope’s trend”, that can predict the complications, is set and determined between POD 2 and 4 and varies from there accordingly.

No matter the actual peak in the CRP value, it was the persistent plateau that defined or predicted the imminent complication reflected by the mean at POD 2–4.

The study of other inflammatory markers (WBC and platelets) on the occurrence of postoperative complications did not demonstrate any statistical significance between patients with and without complications.

One of the weaknesses of our study was its retrospective design. However, measurements for biological inflammatory markers (CRP, platelets, and WBCs) were performed in a systematic way and complications were reported in a prospective database. The major limitation related to the study design was that we evaluated a predictor of complications (CRP) over a shorter period of time (7 postoperative days) compared to the occurrence of the complications themselves, appearing up to 1 month postoperatively. However, such a limitation can actually be employed as a guiding perspective to monitor these patients more thoroughly, even after their discharge home. Serial CRP values (once per week), along with a good bedside examination (chest auscultation, wound examination, abdominal palpation…) might indeed lead to an early diagnosis of an underlying complication, and thus an earlier more effective management.

## Conclusions

The risk of postoperative complications related to cytoreductive surgery associated with HIPEC in patients treated for PC remains significant and diagnosis is often difficult. We report that the kinetics of CRP level, expressed as mean POD 2–4 CRP level, is predictive of postoperative complications in patients treated with CRS plus HIPEC. Patients should be monitored to identify those with persistently high CRP levels expressed as mean POD 2–4 CRP levels.

## Data Availability

The data that support the findings of this study are available on request from the corresponding author. The data are not publicly available due to privacy or ethical restrictions.
